# Large lung mass lesion with spontaneous regression in a patient with IgG4‐related lung disease

**DOI:** 10.1002/rcr2.1075

**Published:** 2022-12-19

**Authors:** Kazuhiko Iwasaki, Tomoyuki Araya, Toshiyuki Kita, Tamami Sakai

**Affiliations:** ^1^ Department of Respiratory Medicine National Hospital Organization Kanazawa Medical Center Kanazawa Japan

**Keywords:** IgG4 related disease, IgG4‐related lung disease, large lung mass lesion, lung cancer, spontaneous regression

## Abstract

IgG4‐related lung disease (IgG4‐RLD) may present with a variety of radiological findings, but large lung mass lesion are rare. Although steroid therapy is strongly recommended for IgG4‐RLD with or without symptoms, respirologists should be aware that some patients may not need steroid therapy.

## Graphical abstract

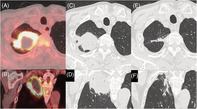

## CLINICAL IMAGE

**FIGURE 1:**
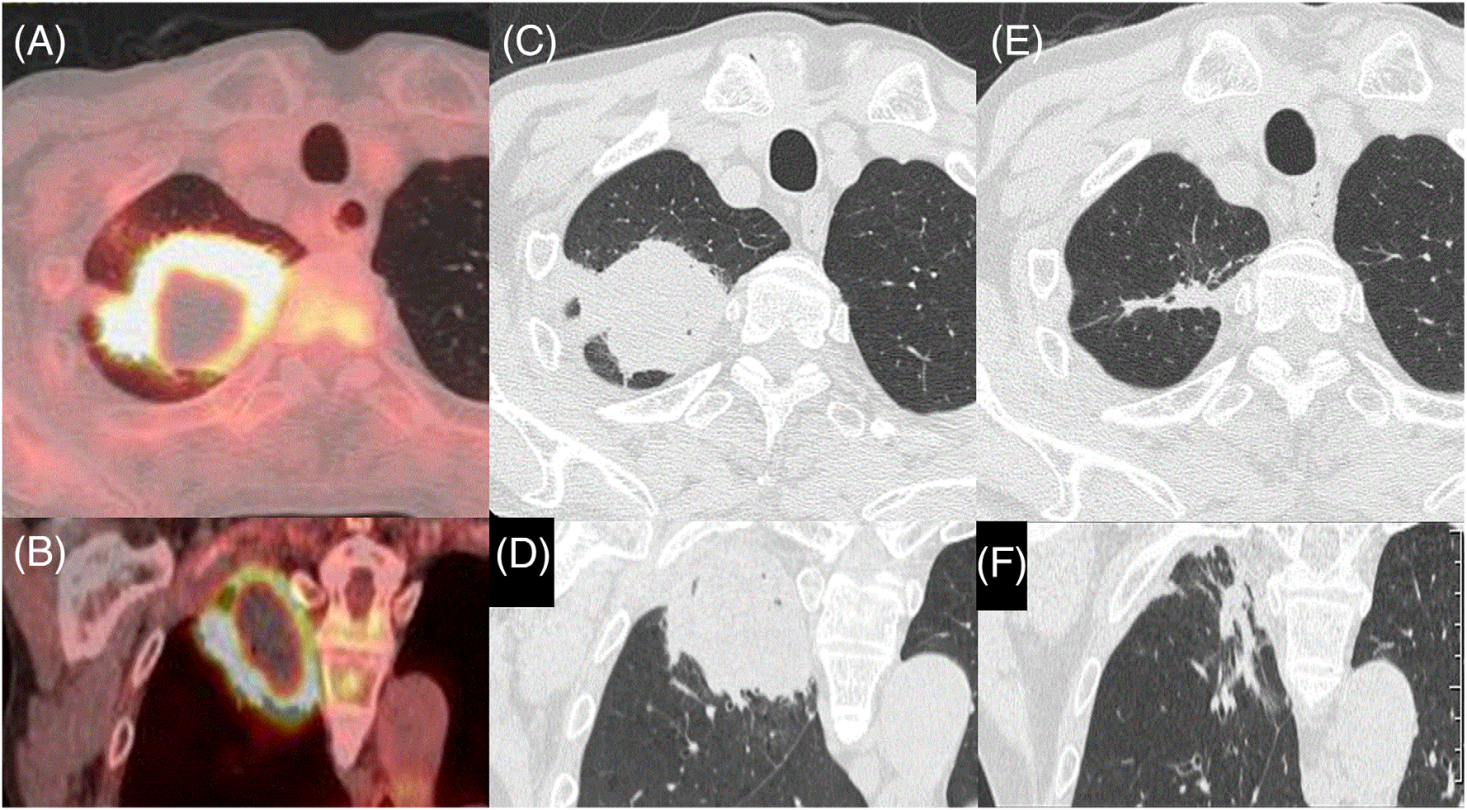
18 F‐fluorodeoxyglucose positron emission tomography/computed tomography (18F‐FDG‐PET/CT) showing a strong accumulation of FDG (maximum standardized uptake value of 12.6) in the right upper lobe of the lung, especially in the margin of the mass lesion (A, B). Chest CT on admission demonstrating a large mass shadow (65 mm in diameter) in the right upper lobe of the lung (C, D). Chest CT 4 months post diagnosis revealing the disappearance of the lung mass lesion (E, F)

**FIGURE 2:**
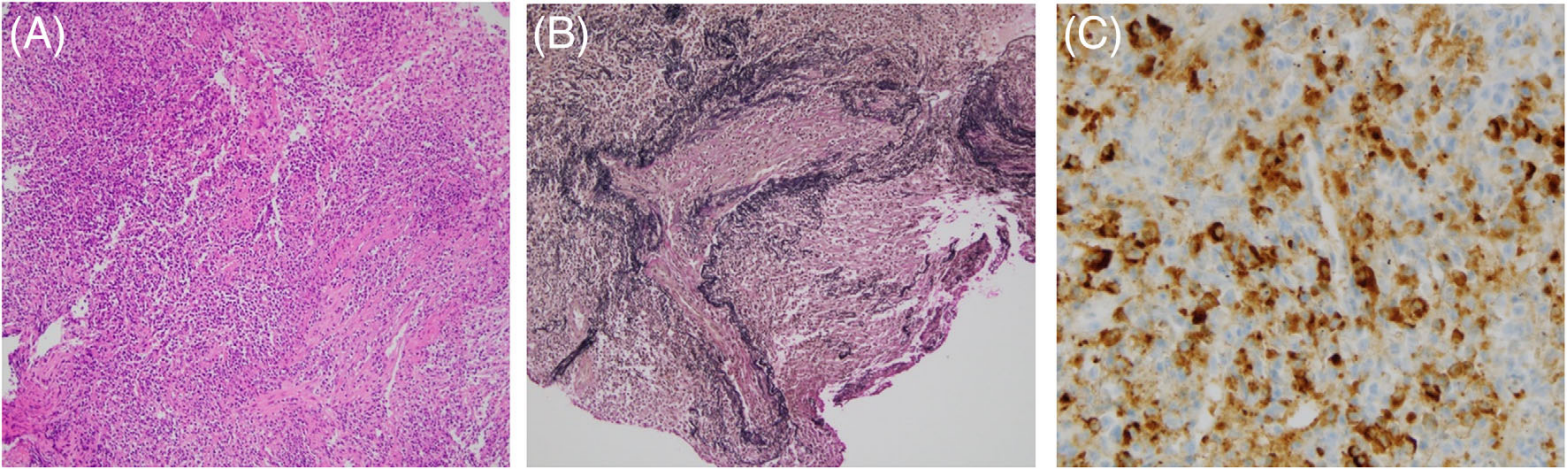
Haematoxylin eosin staining of transbronchial biopsy samples from the right upper lung mass showed inflammatory cells and lymphocyte infiltration (A), while Elastica van Gieson staining revealed obliterative phlebitis (B). Immunohistochemistry showed that average number of IgG4 and IgG positive plasma cells were 118/high‐power field (HPF) (range 113–123/HPF) and 279.5/HPF (range 200–355/HPF), respectively. Average IgG4+/IgG+ ratio of plasma cells was 48.5% (range 31.8–61.5%) (C)

A 74‐year‐old man was admitted to our hospital for suspected lung cancer. He had a history of IgG4‐related pancreatic lesion spontaneously regressing within a month, 6 years ago. 18F‐fluorodeoxyglucose positron emission tomography/computed tomography revealed strong accumulation in the lung mass lesion margin. No other organs showed abnormal accumulations (Figure 1A, B). Laboratory tests showed elevated serum IgG4 (197 mg/dl; normal <135 mg/dl) levels, whereas tumour markers were within normal range. Furthermore, collagen‐vascular disease or infection were not observed. Biopsy samples from the right upper lung mass showed lymphocyte infiltration, obliterative phlebitis and numerous IgG4‐positive plasma cells (Figure 2A–C). In accordance with IgG4‐related lung disease (IgG4‐RLD) diagnostic criteria,^1^ a diagnosis was made based on imaging, serological, pathological findings, and previous history of IgG4‐related disease. Post diagnosis, the large lung mass shrunk remarkably without steroid therapy. Four months, the lesion nearly disappeared (Figure 1E, F).

A large lung mass lesion presenting with spontaneous regression in a short period has not been reported in a patient with IgG4‐RLD. Despite solid lung nodules being reported in 33% of patients with IgG4‐RLD, the frequency of a mass lesion occurrence is not accurately known.^2^ Respirologists should be some IgG4‐RLD may not need steroid therapy.

## AUTHOR CONTRIBUTIONS

Kazuhiko Iwasaki wrote the initial draft of the manuscript and was responsible for drafting and image modification. Kazuhiko Iwasaki, Tomoyuki Araya, Toshiyuki Kita, and Tamami Sakai were directly involved in treatment, critically revised the manuscript, and approved the final version.

## CONFLICT OF INTEREST

None declared.

## ETHICS STATEMENT

Patient consent obtained and the application number of our Ethics Committee is R04‐019.

## Data Availability

No datasets were generated or analyzed for this case report.
